# Investigating the Effective Factors on the Acceptance of Teleorthodontic Technology Based on the Technology Acceptance Model 3 (TAM3)

**DOI:** 10.30476/dentjods.2023.96932.1977

**Published:** 2024-03-01

**Authors:** Fatemeh Yazdanpanahi, Mehraban Shahi, Mehrdad Vossoughi, Nasrin Davaridolatabadi

**Affiliations:** 1 MSc Student in Health Information Technology, Student Research Committee, School of Paramedicine, Hormozgan University of medical sciences, Bandar Abbas, Iran; 2 Dept. of Health Information Management, School of Paramedicine, Hormozgan University of Medical Sciences, Bandar Abbas, Iran; 3 Mental Health Research Center, Psychosocial Health Research Institute (PHRI), Iran University of Medical Sciences, Tehran, Iran; 4 Dept. of Health Information Management, Dept. of Health Information Management, School of Paramedicine, Hormozgan University of Medical Sciences, Bandar Abbas, Iran

**Keywords:** Telemedicine, Dental technology, Technology, Orthodontics

## Abstract

**Statement of the Problem:**

Health information technology is used in dentistry worldwide. Despite the limited specialized resources for providing orthodontic treatment in Iran, the need to examine the technology acceptance model (TAM) seems necessary and is a significant step in the successful acceptance of teleorthodontic technology.

**Purpose:**

The present study has identified and investigated the factors affecting the acceptance of teleorthodontic technology among orthodontists based on the TAM3 with the aim of successful implementation and deployment of this technology.

**Materials and Method:**

In this descriptive-analytical research, 300 Iranian orthodontists who were members of the Iranian Orthodontic Association were selected by census sampling. The data was gathered through a modified and accommodated questionnaire called the acceptance model 3. The validity was confirmed. Moreover, the reliability was calculated based on Cronbach's alpha, which was equal to 0.870. Multiple linear regression analysis was also utilized to investigate the relationships between dependent, independent, and mediator variables. Besides, the final model was designed by the Amos software.

**Results:**

The results of 251 orthodontic specialists proved that subjective norm, job relevance, output quality, results in demonstrability, and job relevance on output quality could significantly affect perceived usefulness. Similarly, the perception of external control was identified to have a significant influence on perceived ease of use. On the other hand, the perceived usefulness does not play a mediating role between perception and subjective norm. Furthermore, perceived usefulness was confirmed as a mediating factor in relationship to both perceived ease of use and behavioral intention.

**Conclusion:**

The findings of the present study revealed valuable scientific evidence to identify and apply the key factors affecting the acceptance and use of modern teleorthodontic technology in Iran. Besides, the structure of the TAM3 was recognized as fruitful and worthwhile for predicting the acceptance of this new technology and also in identifying key effective factors.

## Introduction

Orthodontics is a specialized branch in dentistry including the diagnosis, prevention, and treatment of dental disorders and maxillofacial disorders. Additionally, this branch as a kind of dentistry orthopedics endeavors to boost the growth of the human’s jaw, face, and teeth, and it simultaneously considers how they are connected to each other [ 1 - 2 ]. According to studies, the prevalence of dental-maxillofacial malformations is spreading on a large scale universally [ 3 ]. More than 45% of children aged 2 to 7 years in China, 35.40% in India, 80% in Indonesia, and 88% of those studied in Saudi Arabia have suffered from these dental disorders [ 4 - 7 ].This is while the rate of this anomaly is 87% in Iran [ 8 ]. Thus, the necessity of a decent treatment is noticeable; correspondingly, various conditions and prospects must be considered which are challenging issues in the field [ 9 ].

One of the unavoidable challenges is the necessity of acceptance of the specialized dental services such as orthodontics, especially in rural and remote areas and among developing countries [ 10 - 11 ].In addition, orthodontics is considered an ongoing treatment and besides, early identification and prevention of problem progression is critical. In other words, orthodontics as a treatment requires the constant supervision of an orthodontist to evaluate the effectiveness or adverse effects of the treatment process for early diagnosis and subsequently, preventing the occurrence of serious complications. However, not all periodic visits are necessary and can be avoided by taking precautions. There are several solutions to the challenges [ 12 - 14 ].

 One of the efficient solutions is to provide dental services in today's world by making use of updated technologies, which can be useful by reconsidering the existing procedures to achieve professional goals [ 15 ]. Teleorthodontic means the use of information and telecommunication technology to facilitate the management of orthodontic treatment, which can be beneficial in four areas including teleconsulting, teleeducation, telemonitoring, and telesurgery [ 10 , 16 ] 

This new technology can relate the orthodontists to each other in different geographical locations around the world. Moreover as a tool, it can be used to initiate early evaluation and treatment, create a close relationship between patients and the specialists, use time more efficiently, and be cost-effectiveness [ 1 , 12 ]. Use of dedicated emojis for disadvantaged children, virtual reality, augmented reality, instant messaging, dedicated chat rooms, digitizing files, and dedicated applications in the context of different technologies are used in orthodontic traps [ 17 - 18 ]. However, the use of various technologies makes orthodontic treatment safer, more efficient. Hence, orthodontists have been considered as pioneers in moving towards digitalization and adopting information technology [ 19 ]. However, despite the increasing use of orthodontists of information technology, its practical use and adaptation is directly related to the motivation of specialists. Consequently, in order to predict the success of implementing systems, the technology acceptance models (TAM) is illustrated in details [ 20 ]. 

TAM is a theory of information systems that models how users accept and use technology [ 21 ].The designers of this model state that knowing the goals of end users have a great impact on increasing the future use of technology. In addition, by knowing the reasons for users' goals, we will be able to manipulate factors to strengthen technology acceptance [ 20 ]. In general, the TAM is one of the most common models used to understand the acceptance of new technology [ 22 - 23 ].

Examining the acceptance of teleorthodontic technology based on the TAM3 to determine the motivation, purpose, and ideals of orthodontists is an important step towards acceptance, access, and effective use of orthodontic technology [ 24 ]. Therefore, despite the limited resources for orthodontic services in Iran [ 10 ], the need to consider the acceptance of this technology seems necessary and is a key step toward the successful acceptance of this technology.

## Materials and Method

### Research framework

The present study is a quantitative study and in terms of the research plan, it is an analytical-descriptive one, which was accomplished in 2021. This type of research is considered correlational and purpose-based. It is also a field study because of utilizing a questionnaire. This study was designed to examine the factors and modeling items based on the TAM3 standard framework. Technology adoption models have developed 20 years ago, TAM3 has been developed over the past 20 years, and TAM3 was designed to use complementary elements added to the original model [ 25 ]. TAM3 is more comprehensive in comparison to the previous models namely TAM2 and ease of use model determinants, since TAM3offers a more complete network of determinants and factors in the acceptance and use of information technology regarding the three theoretical formats [ 26 ]. TAM3 standard framework includes four main structures including perceived usefulness, perceived ease of use, behavioral intention, and use behavior. According to this model, perceived usefulness and perceived ease of use are variables that illustrate the relationship between a dependent (behavioral intention) and independent variable. Six independent variables, including subjective norm, image, job relevance, output quality, productivity, and voluntaries can affect perceived usefulness. Also, six independent variables, including self-efficacy, perception of external control, computer anxiety, perceived enjoyment, computer playfulness, and objective usability can affect perceived ease of use [ 26 - 27 ]. As a result, the research hypothesis and summary of results are as following.

### Research tools

The theoretical part of the research has been done by using the library method and reviewing of valid internet databases such as PubMed, ProQuest, ISI, Scopus, Science Direct, Springer, and Google scholar search engine. Iranian databases such as SID, Magiran, and ISC were used to localize the questionnaire questions as the data collection procedures. The data collection tool is a questionnaire. This questionnaire is divided into two parts; the first part includes general and demographic questions, including gender, and age. The second part consists of specific questions according to the TAM3 standard framework. Thus, the research collection tool comprises of 51 questionnaire questions in the form of 15 localized sections, which covers all aspects related to the acceptance of this novel technology. The questionnaire was uploaded and responded by the participants online (via social networks such as WhatsApp, Instagram, and email) in order to collect the views of the respondents. Finally, all aspects of orthodontists' attitudes in the pre-implementation phase of the technology were measured by using TAM3.The measurement tool related to teleorthodontic acceptance was a 5-point Likert scale. The validity of the questionnaire was confirmed by seven experts, including five specialists in health information management and medical informatics and two orthodontists. In addition, the translation of the questionnaire into Persian was reviewed by two English language experts who were fully aware of the content of the questionnaire. In addition, the content validity of this questionnaire has been investigated by similar studies [ 27 - 30 ] .The reliability of this questionnaire was tested using Cronbach's alpha (coefficient) test and its value was 0.870, which indicates the high reliability of the present questionnaire.

### Research environment and participants

This study used a quantitative design in which the data were gathered through objective measurement tools and then were analyzed statistically making use of SPSS. In order to achieve the objectives, the participants were chosen via census sampling procedure from the Research Community of Orthodontists or to be more accurate, from the Iranian Orthodontic Association. At the beginning of the study, the available participants were considered to be 300 orthodontists. The census sampling method as a non-probability sampling was used in order to create a theory or to confirm existing theories for a special stratum of the statistical population, thus, a census sampling method was used to study every available unit in the population. It is also known as a complete enumeration, meaning a complete count of the target sample.

### Data collection

This study was approved by the Ethics Committee of Hormozgan University of Medical Sciences (IR.HUMS. REC.1400.356.). All members of the sample community were invited and a questionnaire was provided to them online. Participation in this study was voluntary and each person had the freedom to leave the study without any consequences.

### Statistics

The data were collected after completing and returning the questionnaires, then, were entered meticulously in version 23 of SPSS software. Descriptive indicators such as frequency, frequency percentage, cumulative frequency percentage, mean and standard deviation were calculated and determined. In addition, the multiple linear regression method was used to illuminate on the relationship between variables and the results were analyzed. Then, in Amos software, data from SPSS were called and the final model was designed and presented.

### Setting and contribution

A total of 300 questionnaires were distributed among the target population, of which 251 valid questionnaires were returned. The response rate of 83.6% was calculated. 40.45% of the participants were women and 50.54% were men. 27.2% of the participants were under 40, 67.5% were between 40 and 50, and 8%over 50 year.

### Statistical Methods

**Figure 1:**
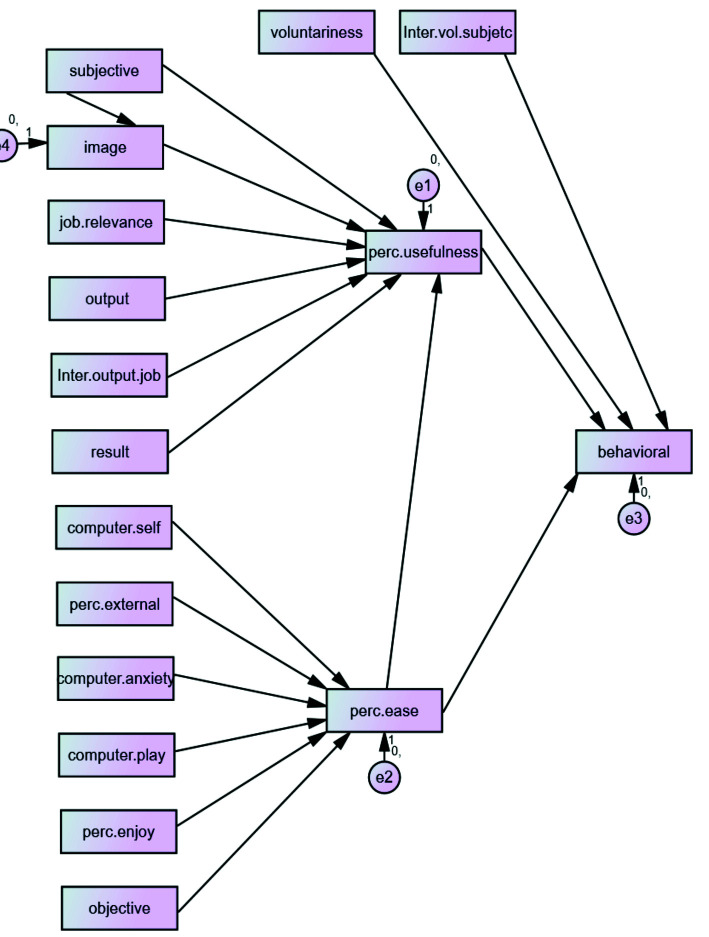
The tested model Subjective= subjective norm, image= image, Job. Relevance=job relevance, Output= output quality, Inter.output.job= output quality * job relevance, Result= result demonstrability, Computer. self= computer efficacy, perc. External=perceptions of external control, Computer anxiety=computer anxiety, computer. Play= computer playfulness, perc.enjoy=perceived enjoyment, objective= objective usability, perc. Usefulness perceived usefulness, perc.ease= perceived ease of use, behavioral= behavioral intention, voluntariness= voluntariness, inter.vol.subject=subjective norm* voluntariness

Path analysis was used to check the appropriateness of TAM3 model hypotheses. To this end, IBM SPSS AMOS version 22 software was used. In all hypothesis tests, the equaled level was considered equal to 0.05. The tested model is shown in Figure 1.

### Participants

The present study included 251 (137 men and 114 women) orthodontic specialists who have completely responded to the questionnaire. 69 people (27.2%) were under 40 years old, 162 people (64.5%) were 40-50 years old, and 20 people (8%) were over 50 years old.

## Results

### Hypotheses testing results

Tables 1 and 2 show the results of the direct and indirect effects of the variables in the model, respectively. Examining the hypotheses Ha1-Ha5 proved that among the five sub-set variables of perceive usefulness, only job relevance, output quality, and result demonstrability variables have a significant effect on perceived usefulness. Examining the first five hypotheses of series c indicated that all the three mentioned variables have a significant direct effect on behavioral intention. Based on the hypothesis, the perceived variable was expected to be a mediator, however, the direct effect of perceived usefulness on behavioral intention and the indirect effects of the five mentioned variables on behavioral intention were not significant (hypotheses H1-H5 in Table 2).

**Table 1:** Study’s hypothesis and summary of results

Hypothesis	*p*	Agreement
Ha1: Subjective Norm→A	0.469	✗
Ha2: Image→A	0.623	✗
Ha3: Job Relevance→A	0.001	✓
Ha4: Output Quality→A	0.001	✓
Ha5: Result Demonstrability→A	0.028	✓
Ha6: (Job Relevance*Output quality)→A	0.006	✓
Hb1: Computer Self-efficacy→B	0.099	✗
Hb2: Perceptions of External Control→B	<0.001	✓
Hb3: Computer Anxiety→B	0.067	✗
Hb4: Computer Playfulness→B	0.611	✗
Hb5: Perceived Enjoyment→B	0.090	✗
Hb6: Objective Usability→B	0.062	✗
Hc1: Subjective Norm→C	0.308	✗
Hc2: Image →C	0.069	✗
Hc3: Job Relevance →C	0.001	✓
Hc4: Output Quality →C	0.007	✓
Hc5: Result Demonstrability →C	0.037	✓
Hc6: Computer Self-efficacy →C	0.609	✗
Hc7: Perceptions of External Control →C	0.074	✗
Hc8: Computer Anxiety →C	0.001	✓
Hc9: Computer Playfulness →C	0.021	✓
Hc10: Perceived Enjoyment →C	0.216	✗
Hbc11: Objective Usability →C	0.132	✗
Hd1: A→C	0.181	✗
Hd2: B → C	0.960	✗
Hd3: B → A	0.023	✓
He1: Voluntariness→C	0.290	✗
He2: (Subjective Norm*Voluntariness) →C	0.317	✗
He3: Subjective Norm→Image	0.001	✓

A: Perceived usefulness, B: Perceived ease of use, C: Behavioral Intention

**Table 2:** Testing hypotheses of indirect effects in the model

Hypothesis	*p*	Agreement
H1: Subjective Norm→ Image→ A	0.549	✗
H2: Subjective Norm→ A→ C	0.259	✗
H3: Image→ A→ C	0.523	✗
H4: Job Relevance→ A→ C	0.001	✓
H5: Output Quality → A→ C	0.001	✓
H6: Result Demonstrability → A→ C	0.024	✓
H7: Perceptions of External Control→ BA	0.016	✗
H8: Computer Self-efficacy→ BA	0.060	✓
H9: Perceptions of External Control→ BA	0.017	✓
H10: Computer Anxiety→ BA	0.054	✗
H11: Computer Playfulness→ BA	0.469	✗
H12: Perceived Enjoyment→ BA	0.068	✗
H13: Objective Usability→ BA	0.047	✓
H14: Perceptions of External Control→ (A,B)→C	0.075	✗
H15: Computer Self-efficacy→ (A,B)→C	0.102	✗
H16: Computer Anxiety→ (A,B)→C	0.078	✗
H17: Computer Playfulness→ (A,B)→C	0.478	✗
H18: Perceived Enjoyment→ (A,B)→C	0.106	✗
H19: Objective Usability→ (A,B)→C	0.078	✗
H20: B → A→ C	0.018	✓

A: Perceived usefulness, B: Perceived ease of use, C: Behavioral Intention

Thus, the Perceived usefulness was not a mediator and had no influence on the relationship between behavioral intention and perceived subgroup variables. Examining the hypothesis Ha6 revealed that there is a significant interaction between the predictive variables of job relevance and output quality with perceived usefulness.

In other words, the output quality variable is a moderator in the relationship between job relevance and perceived usefulness.

**Figure 2:**
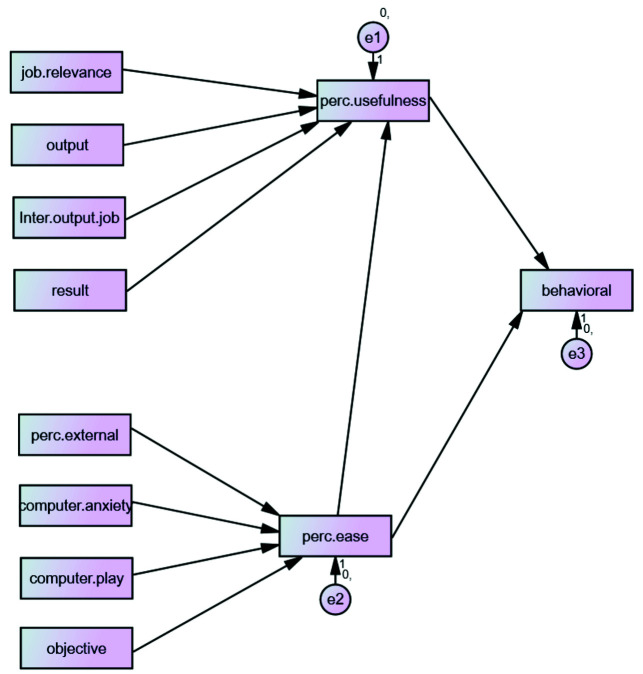
Key factors affecting on behavioral intention Job. Relevance=job relevance, Output= output quality, Inter.output.job= output quality * job relevance, Result= result demonstrability, Computer.self= computer efficacy, perc.external=perceptions of external control, Computer. anxiety=computer anxiety, computer. Play= computer playfulness, objective= objective usability, perc.usefulness perceived usefulness, perc.ease= perceived ease of use, behavioral= behavioral intention.

The results of the series b hypothesis test showed that there exists one significant relationship between perceived external control and the perceived ease of use. On the other hand, the other five variables of the superset of perceived ease of use showed no significant effect on this variable. Examining hypotheses Hc6-Hc11 shows a significant direct relationship between computer anxiety and computer playfulness with behavioral intention. Thus, perceived ease has a significant direct effect on perceived usefulness and the indirect effect of perceived variables. External control and objective usability on perceived usefulness were significant (Table 2), therefore, it can be concluded that the perceived ease of use variable has a relationship between objective usability and perceived usefulness. It mediates perceived usefulness completely and also partially mediates the relationship between the perception of external control and perceived usefulness partially. Nevertheless, this variable is a mediating variable for the relationship between other variables in which the perceived usefulness was not. There is no indirect significant effect between the six variables of the perceived ease of use subset and behavioral intention. Subsequently, none of these variables can be changed through perceived usefulness and perceived ease of use (as mediators) and none of them were related to behavioral intention. Perceived usefulness and perceived ease of use had no significant direct relationship with behavioral intention. However, there is a significant indirect relationship between perceived ease of use and behavioral intention through the perceived usefulness (as a full mediator). Moreover, the assumption of moderator or interaction of voluntariness variable in the relationship between subjective norm and the behavioral intention was rejected. Figure 2 shows key affecting factors on behavioral intention. Table 1 demonstrates a summary of the hypothesis results.

## Discussion

The main findings of TAM3 including the relationship between perceived usefulness, perceived ease of use, and behavioral intention to adopt the system were remained unconfirmed.

The findings of the current study indicated that the teleorthodontic technology was considered to be easy by its intended users, concluding that it does not require much effort to handle this novel technology. Moreover, there is a direct relationship between the perceived usefulness and the behavioral intention, which means they simultaneously increase. The results also confirm the effect of perceived ease of use on perceived usefulness. Furthermore, the results’ demonstrability has a mediating effect on the perceived usefulness. As a result, it can be stated that these independent variables are determinants and predictors of perceived usefulness. This research is consistent with the study of Zhu *et al*. [ 31 ].

Ebn Hosseini *et al*. [ 28 ], Zhang *et al*. [ 32 ] Su YY *et al*. [ 33 ] and De Angelis *et al*. [ 34 ] also confirm the effect of job relevance on perceived usefulness. Furthermore, the study by Lee SS *et al*. [ 35 ] and Chen *et al*. [ 36 ] confirm the effectiveness of output quality on perceived usefulness. The research done by Usmanova *et al*. [ 30 ] confirms the effect of the results' demonstrability on the perceived usefulness. Nadri *et al*. [ 37 ] and Ebrahimi *et al*. [ 38 ] revealed that there were significant relations between job relevance and output quality with perceived usefulness. However, according to the results of the study, there is no relationship between perceived usefulness and behavioral intention. As a result, these factors cannot be considered to be effective factors in the adoption of teleorthodontic technology. In addition, subjective norms and images do not affect perceived usefulness. Image refers to the degree to which a person perceives that the use of innovation and new systems will increase and improve his/her position in his/her social system [ 26 ]. Moreover, subjective norm refers to the degree to which a person perceives that the majority of people who are important to him/her in terms of the profession are willing to use the new system [ 26 ]. A possible reason for the lack of influence of subjective norms on perceived usefulness can be the lack of influence of social pressure and thoughts on orthodontic specialists. In addition, the lack of impact on the image can be attributed to the lack of knowledge of teleorthodontics technology and the advantages of this technology by orthodontic specialists.

Perception of external control is considered the only influencing factor on perceived ease of use as a mediating variable on behavioral intention. As a result, it is considered a key factor in the acceptance of teleorthodontic technology among orthodontic specialists. This finding is in line with the studies of Domingos *et al*. [ 39 ], Isernia *et al*. [ 43 ], and Ho *et al*. [ 40 ].

Computer self-efficacy, computer anxiety, perceived enjoyment, computer playfulness, and objective usability have been identified as factors that have no effect on perceived ease of use and thus have no effect on behavioral intention. Computer self-efficacy can be defined as the degree to which a person feels himself / herself as skillful enough in accomplishing certain tasks while using computers [ 41 ]. In addition, computer anxiety is the degree to which someone is concerned about or even fears when they confront the opportunity to use computers [ 42 ]. Computer playfulness can be described as the degree of perceived spontaneity while utilizing a technology [ 41 ]. Perceived enjoyment refers to the performance-related consequences of using a novel system and the extent to which using the system is perceived as enjoyable [ 42 ]. Objective usability is determined by comparing the amount of time spent by an expert using a traditional method with the time spent by a novice performing a task using a new technology [ 42 ]. Accordingly, it can be concluded that orthodontic specialists have no desire to replace the practical process with computer systems. In addition, these specialists understand the pleasure of working with teleorthodontics. However, they prefer the time saved by the traditional system. These results are in line with the research of Chang *et al*. [ 27 ], Chen *et al*. [ 36 ], Isernia *et al*. [ 43 ], Ho *et al*. [ 40 ], Cengiz *et al*. [ 42 ], Mansourzadeh *et al*. [ 44 ]. Eventually, lack of implementation and the beneficial use behavior items in the questionnaire can be regarded as the limitations of this study, which in turn is due to the absence of practical application of similar technology. The findings of the present study provide valuable scientific evidence for key factors influencing the implementation and acceptance of teleorthodontic technology among orthodontic specialists for health care providers, administrators, and policymakers. As a result, we will have strategies and policies to successfully implement and facilitate the adoption of teleorthodontics among orthodontic professionals.

## Conclusion

Based on the findings, orthodontic specialists are not familiar with teleorthodontics technology and the advantages of this technology in practice. According to TAM 3, the ease of use of teleorthodontics technology is effective on the understanding of the usefulness of this technology by the end users, which can lead to an increase in the practical future use of this technology. Moreover, based on the results, it can be suggested that the benefits of this technology are widespread among the users. In conclusion, this model can support software designers and other researchers, because it has decent indicators, which can be used in the successful implementation and investigation of factors affecting the use of teleorthodontic technology by the practical implementation of the technology in Iran. 
